# OVERVIEW OF NIA MISSION AND RESEARCH

**DOI:** 10.1093/geroni/igae098.0783

**Published:** 2024-12-31

**Authors:** Richard Hodes

**Affiliations:** National Institute on Aging, Baltimore, Maryland, United States

## Abstract

Dr. Hodes will provide an overview of NIA’s structure and mission, in addition to discussing research foci from across the Institute’s scientific divisions.

